# The Protective Effect of Oral Application of Corni Fructus on the Disorders of the Cornea, Conjunctiva, Lacrimal Gland and Retina by Topical Particulate Matter 2.5

**DOI:** 10.3390/nu13092986

**Published:** 2021-08-27

**Authors:** Hyesook Lee, Min Yeong Kim, Seon Yeong Ji, Da Hye Kim, So Young Kim, Hyun Hwangbo, Cheol Park, Su Hyun Hong, Gi-Young Kim, Yung Hyun Choi

**Affiliations:** 1 Department of Biochemistry, College of Korean Medicine, Dong-Eui University, Busan 47227, Korea; 14769@deu.ac.kr (H.L.); ilytoo365@deu.ac.kr (M.Y.K.); 14602@deu.ac.kr (S.Y.J.); believe0402@naver.com (D.H.K.); 14731@deu.ac.kr (S.Y.K.); hongsh@deu.ac.kr (S.H.H.); 2 Anti-Aging Research Center, Dong-Eui University, Busan 47340, Korea; 3 Korea Nanobiotechnology Center, Pusan National University, Busan 46241, Korea; hbhyun2003@naver.com; 4 Department of Molecular Biology, College of Natural Sciences, Dong-Eui University, Busan 47340, Korea; parkch@deu.ac.kr; 5 Department of Marine Life Science, Jeju National University, Jeju 63243, Korea; immunkim@jejunu.ac.kr

**Keywords:** Corni Fructus, cyclosporine A, dry eye disease, goblet cells, lutein, particulate matter 2.5, retinal ganglion cells, tear production

## Abstract

Particulate matter 2.5 (PM_2.5_) may aggravate dry eye disease (DED). Corni Fructus (CF), which is fruit of *Cornus officinalis* Sieb. et Zucc., has been reported to have various beneficial pharmacological effects, whereas the effect of CF on the eye is still unknown. Therefore, in this study, we investigated the effect of oral administration of water extract of CF (CFW) on the eye, hematology, and biochemistry in a DED model induced by topical exposure to PM_2.5_. Furthermore, the efficacy of CFW compared with cyclosporine (CsA), an anti-inflammatory agent, and lutein, the posterior eye-protective agent. Sprague-Dawley rats were topically administered 5 mg/mL PM_2.5_ in both eyes four times daily for 14 days. During the same period, CFW (200 mg/kg and 400 mg/kg) and lutein (4.1 mg/kg) were orally administered once a day. All eyes of rats in the 0.05% cyclosporine A (CsA)-treated group were topically exposed to 20 μL of CsA, twice daily for 14 days. Oral administration of CFW attenuated the PM_2.5_-induced reduction of tear secretion and corneal epithelial damage. In addition, CFW protected against goblet cell loss in conjunctiva and overexpression of inflammatory factors in the lacrimal gland following topical exposure to PM_2.5_. Furthermore, CFW markedly prevented PM_2.5_-induced ganglion cell loss and recovered the thickness of inner plexiform layer. Meanwhile, CFW treatment decreased the levels of total cholesterol and low-density lipoprotein cholesterol in serum induced by PM_2.5_. Importantly, the efficacy of CFW was superior or similar to that of CsA and lutein. Taken together, oral administration of CFW may have protective effects against PM_2.5_-induced DED symptoms via stabilization of the tear film and suppression of inflammation. Furthermore, CFW may in part contribute to improving retinal function and lipid metabolism disorder.

## 1. Introduction

Air pollution is a serious health issue and consists of thousands of solid particles, gases, and liquid droplets [1]. Among the various biologically toxic substances of air pollutants, particulate matter 2.5 is considered the most important harmful substrate of health threat [2]. PM_2.5_ refers to particulate matter with diameter less than 2.5 μm [2]. Several epidemiological and biochemical studies have shown that chronic or acute exposure to PM_2.5_ may be attributed to dry eye disease (DED), also known as keratoconjunctivitis sicca [3,4,5,6,7]. DED is a multifactorial disease of the ocular surface that involves tear film instability, hyperosmolarity, inflammation, and damage to the ocular surface [8,9]. Some studies have reported that people with exposure to severe air pollution often experienced symptoms of DED [6,10,11]. DED symptoms can include foreign body sensation, redness, stinging, pain, and burning on the eyes [6,10,11]. In rodents, studies have recently reported that topical exposure to PM_2.5_ results in the pathological events of DED, and these ocular changes are similar to those in humans [12,13,14]. Some cell-based studies have demonstrated that PM_2.5_ induces cytotoxicity, DNA damage, inflammation, and wound healing suppression in corneal epithelial and conjunctival epithelial cells [15,16,17]. Although air pollution is closely linked to pathogenesis of the ocular system, studies on the harmful effect of PM_2.5_ on the eye and the development of a therapeutic agent are still in the early stage.

The Oriental herbal medicine Corni Fructus (CF), which is fruit of *Cornus officinalis* Sieb. et Zucc., has been widely used to treat kidney deficiency, dizziness, spermatorrhea, pain, and hypertension for over 2000 years in eastern Asia [18,19]. Numerous studied have reported that extract of CF has various pharmacological benefits, including anticancer, anti-inflammation, neuroprotection, and anti-oxidative effects [20,21,22,23,24]. In addition, we previously demonstrated that CF inactivated 5α-reductase and androgen receptor, consequentially resulting in suppression of testosterone propionate-induced benign prostatic hypertrophy [25]. More recently, it has been reported that CF protected human HaCaT keratinocytes against PM_2.5_-induced oxidative stress through suppression of Ca^2+^ accumulation and apoptosis [26]. Furthermore, several reports suggested that bioactive components from CF, such as terpenoids, flavonoids, tannins, and furans, exhibit anti-inflammatory, anti-oxidative, and anti-apoptotic activities [27,28,29]. Recently, we also demonstrated that loganin and morroniside, major iridoid glycosides isolated from CF, suppressed lipopolysaccharide-induced inflammation and oxidative response through activation of the nuclear factor erythroid 2-related factor 2/heme oxygenase-1 signaling pathway in RAW264.7 macrophages [27,30]. Despite reports that CF has various beneficial pharmacological effects, the effect of CF on the eye is still unknown. Therefore, in this study, we investigated the effect of oral administration of water extract of CF (CFW) on histological changes in the eye, including the cornea, conjunctiva, lacrimal gland, and retina, and on the changes in hematology and biochemistry in dry eye rat models induced by topical exposure to PM_2.5_. Furthermore, we evaluated the efficacy of CFW compared with lutein and cyclosporine A (CsA). Lutein is one of the xanthophyll carotenoids and has eye-protective properties [31], and CsA, an anti-inflammatory and a T cell immunomodulatory agent, is used to suppress ocular surface inflammation and improve tear film dynamics [32,33].

## 2. Materials and Methods

### 2.1. Preparation of PM_2.5_ and Treatments

The National Institute of Standards and Technology (NIST, Gaithersburg, MD, USA) SRM 1650 b standard diesel PM_2.5_ and spermidine were purchased from Sigma-Aldrich Chemical Co. (St. Louis, MO, USA). A 25 mg/mL stock solution of PM_2.5_ was prepared in dimethylsulfoxide (Invitrogen, Carlsbad, CA, USA) and diluted to the 5 mg/mL of PM_2.5_ in distilled water. The dried fruits of CF were provided by Gurye Sansuyu Farming Association Corporation (Jeollanam-do Province, Republic of Korea), and CFW was prepared according to a previous study [25]. CFW was diluted to the required concentrations in distilled water immediately before use. Lutein was obtained from Solgar (Leonia, NJ, USA). Topical CsA (0.05% Cyporin N^®^ eye drops) was obtained from Taejoon Pharma Co., Ltd. (Seoul, Korea).

### 2.2. Animals and Experimental Procedures

Animal care and all experiments were performed in accordance with the Guide for Animal Experimentation of Dong-eui University with the approval of the Institutional Animal Care and Use Committee (IACUC approval No. R2019-005). Six-week-old female Sprague-Dawley (SD) rats were obtained from Samtako Bio Korea Co. (Osan, Korea) and housed in a semi-pathogen-free facility with a temperature of 22–24 °C, relative humidity of 50–60%, and 12 h light/12 h dark cycles. After acclimatization for a week, the rats were randomly divided into six groups: untreated control group (Control, *n* = 5); PM_2.5_-induced DED group (DED, *n* = 5); PM_2.5_ with 200 mg/kg CFW group (CFW 200, *n* = 5); PM_2.5_ with 400 mg/kg CFW group (CFW 400, *n* = 5); PM_2.5_ with 4.1 mg/kg lutein group (Lutein, *n* = 5), and PM_2.5_ with CsA group (CsA, *n* = 5). DED was induced by topically administering 20 μL of 5 mg/mL PM_2.5_ in both eyes four times daily for 14 days, except for the control group. During the same period, CFW and lutein were administered orally once a day for 14 days. All eyes of rats in the CsA group were topically exposed to 20 μL of CsA, twice daily for 14 days. Body weight was measured at baseline and on day 14. The rats in all the groups were euthanized on day 14. After midline thoracotomy, whole blood and serum were prepared as previously described [34]. The left ventricle was catheterized using a 23-gage butterfly needle (Becton Dickinson, Franklin Lakes, NJ, USA), the femoral artery was incised and perfused with sterile saline at the rate of 10 mL/min for 3 min. After perfusion, the organs were immediately excised and weighed. The eyes and adnexa were dissected and fixed in 10% formalin for histological and immunohistochemical analyses.

### 2.3. Hematological and Biochemical Analysis

Red blood cell (RBC) count, white blood cell (WBC) count, hematocrit, hemoglobin levels, mean corpuscular volume (MCV), mean corpuscular hemoglobin (MCH) levels, MCH concentration (MCHC), and platelet count were measured using a Sysmex XN-9000 analyzer (Sysmex Corporation, Kobe City, Hyogo Prefecture, Japan). Serum alanine aminotransferase, aspartate aminotransferase, alkaline phosphatase, blood urea nitrogen, creatinine, and lipid profiles were analyzed using Cobas 8000 C702 chemistry analyzer (Roche, Mannheim, Germany).

### 2.4. Tear Production

On days 0, 7, and 14 post-treatment, tear volume was measured using phenol red tear threads (Jingming Ltd., Tianjin, China) [35]. Briefly, threads were inserted into the lateral canthus of the lower eyelid for 1 min. The length of the red portion of the threads was measured, and the tear volume was expressed in millimeters (mm).

### 2.5. Hematoxylin and Eosin (H&E) Staining

Fixed eyeballs and lacrimal glands were embedded in paraffin and cut into 5 μm sections using a microtome (Leica RM2245, Leica Biosystems, Heidelberg, Germany). The sections were deparaffinized, hydrated, and stained with hematoxylin and eosin (YD Diagnostics Co., Yongin, Korea) The stained slides were observed using the EVOS FL Auto 2 imaging system (Thermo Fisher Scientific, Waltham, MA, USA).

### 2.6. Periodic Acid-Schiff (PAS) Staining

Paraffin-embedded sections of the entire globe were cut into 5 μm thickness, and stained using a PAS kit (Sigma-Aldrich Chemical Co., St. Louis, MO, USA) according to the manufacturer’s protocol. Images of violet PAS-positive goblet cells were acquired using a microscope (Carl Zeiss, Oberkochen, Germany) at the Core Facility Center for Tissue Regeneration (Dong-eui University, Busan, Korea).

### 2.7. Immunohistochemistry 

Sections 5 μm thick of the lacrimal gland were deparaffinized, hydrated, processed in antigen retrieval solution (Abcam Inc., Cambridge, UK), and exposed to 3% H_2_O_2_ solution (Sigma-Aldrich Chemical Co., St. Louis, MO, USA) for 30 min. For immunohistochemical analysis, the slides were incubated with primary antibodies against cluster of differentiation 4 (CD4; Novus Biologicals, Littleton, CO, USA), interleukin-17 (IL-17; Abcam Inc., Cambridge, UK), and tumor necrosis factor alpha (TNF-α; Abcam Inc., Cambridge, UK) for 1 h. Subsequently, the sections were incubated with secondary antibodies (DAKO Corp, Glostrup, Denmark) for 40 min, followed by probing with diaminobenzidine chromogen, and counterstained with Mayer’s hematoxylin (YD Diagnostics Co., Yongin, Korea). The stained slides were photographed using an imaging system (Thermo Fisher Scientific). The quantitative analysis of histological staining for CD4, IL-17, and TNF-α was performed using the “threshold tool” of ImageJ^®^ (National Institutes of Health, Bethesda, MD, USA). 

### 2.8. Statistical Analysis

Data are presented as the mean ± standard deviation. One-way analysis of variance (ANOVA) and post hoc analyses were performed for comparisons between groups using GraphPad Prism 5.03 (GraphPad Software Inc., La Jolla, CA, USA). Statistical significance was set at *p* < 0.05.

## 3. Results

### 3.1. Effect of CFW on the Physiological Changes in PM_2.5_-Exposed Sprague-Dawley (SD) Rats

Body weight was measured in all groups at 0 and 14 days after topical exposure to PM_2.5_, with or without treatment. As shown in Table 1, no significant differences were observed in body weight gain and organ weight between the normal group and PM_2.5_-exposed groups.

### 3.2. Effect of CFW on the Changes of Hematological, Biochemical, and Lipid Profiles in PM_2.5_- Exposed SD Rats

The hematological analysis results showed no differences among the groups (Table 2). In addition, no biochemical abnormalities were observed among the groups. However, the levels of total cholesterol (TC) were markedly increased to 74.47 mg/dL following topical exposure to PM_2.5_, and the levels significantly decreased to control levels after topical administration of 400 mg/kg CFW, which was similar to the levels observed after CsA topical administration. Moreover, low-density lipoprotein cholesterol (LDL-C) levels were also significantly upregulated in the control group, and the levels were downregulated following 200 mg/kg and 400 mg/kg CFW administration. However, oral administration of lutein and topical administration of CsA did not improved serum LDL-C levels. Meanwhile, the levels of high-density lipoprotein cholesterol, triglyceride (TG), and free fatty acid were not different among the groups.

### 3.3. Effect of CFW on Tear Secretion after Topical Exposure to PM_2.5_ in SD Rats

**Figure 1:**
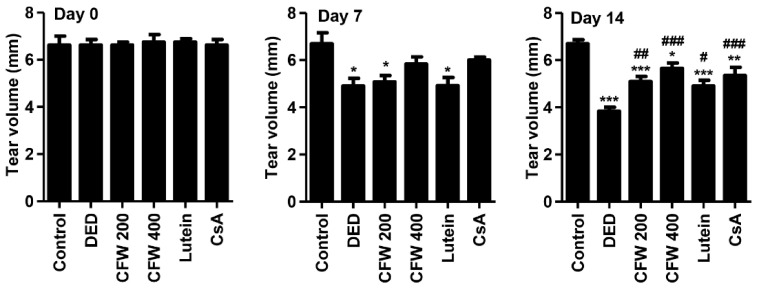
Effect of oral supplements of CFW on tear secretion after topical exposure to PM_2.5_ in SD rats. On days 0, 7, and 14, tear volume was determined using phenol red tear threads and the length of color-changed thread was measured. The data are expressed as the means ± standard deviation (*n* = 5). * *p* < 0.05, ** *p* < 0.01, and *** *p* < 0.001 compared to the control group. # *p* < 0.05, ## *p* < 0.01, and ### *p* < 0.001 compared to the PM_2.5_-induced *DED* group.

We investigated the effect of the oral administration of CFW on the changes in tear production in PM_2.5_-applied SD rats. On days 0, 7, and 14, the tear volume was measured using phenol red tear threads. At day 0, no significant difference was observed in tear production among all groups (Figure 1). During the entire study period, the control group showed no significant difference in tear production. At day 7, tear volume was markedly suppressed in the PM_2.5_-treated DED group (4.90 ± 1.14 mm) compared with that in the control group (6.70 ± 1.05 mm). Although the CFW 200 group (5.08 ± 0.66 mm) and the CFW 400 group (5.83 ± 0.75 mm) showed no significant difference in tear volume compared with the DED group, the tear production gradually increased in the CFW 400 group. At day 14, PM_2.5_ treatment greatly suppressed tear secretion to 3.83 ± 0.61 mm, but oral supplements of 200 mg/kg CFW and 400 mg/kg CFW significantly increased tear production to 5.10 ± 0.86 mm and 5.65 ± 0.71 mm, respectively. Meanwhile, tear production in the lutein group and CsA group was also markedly enhanced compared to that in the DED group at day 14 after treatment.

### 3.4. Effect of CFW on Detachment of Corneal Epithelium in PM_2.5_-Induced DED Rat Model

**Figure 2:**
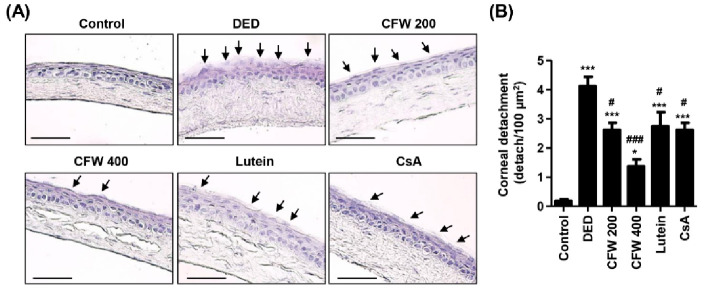
Effect of oral supplements of CFW on the detachment of corneal epithelium in PM_2.5_-induced DED rats. (**A**) Representative images of H&E-stained images of corneal sections (*n* = 4). Black arrows indicate the detached and swollen epithelium. Scale bar, 50 μm. (**B**) The numbers of the detached corneal epithelium of 100 μm^2^ in five different sections were counted. The data are expressed as the means ± standard deviation (*n* = 10). * *p* < 0.05 and *** *p* < 0.001 compared to the control group. # *p* < 0.05 and ### *p* < 0.001 compared to the PM_2.5_-induced DED group.

To investigate whether topical application of PM_2.5_ changes the corneal epithelium and the effect of CFW on PM_2.5_-mediated epithelium alteration, we performed H&E staining. Figure 2A shows that the detachment and swelling of corneal epithelium were more frequently observed in the DED group; however, CFW 200, CFW 400, lutein, and CsA treatments were markedly protect against PM2.5-induced the alteration. Figure 2B shows that the quantitative values of the detached epithelium are indicated as number per 100 μm^2^. Topical exposure to PM_2.5_ markedly enhanced the detachment of corneal epithelium to 4.13 ± 0.63/100 μm^2^. However, the population of detachment epithelium was markedly decreased to 2.63 ± 0.48/100 μm^2^ in the CFW 200 group. Additionally, the detachment of corneal epithelium in the CFW 400 group was greatly suppressed to 1.38 ± 0.47/100 μm^2^ compared with that in the DED group. Meanwhile, the efficacy of administration of lutein and CsA on the corneal epithelium detachment was similar, and the levels were suppressed. This result indicated that oral administration of CFW had a protective effect on the detachment of corneal epithelium by topical exposure to PM_2.5_, and its efficacy was superior to that of lutein and CsA.

### 3.5. Effect of CFW on Conjunctival Goblet Cell Population in PM_2.5_-Induced DED Rat Model

**Figure 3:**
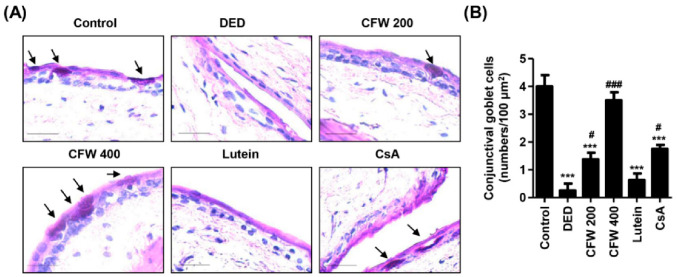
Effect of oral supplements of CFW on conjunctival goblet cell population in PM_2.5_-induced DED rats. (**A**) Representative images of PAS-stained images of conjunctival sections (*n* = 4). Black arrows indicate the PAS-stained goblet cells, which appeared a strong violet color. Scale bar, 25 μm. (**B**) The numbers of goblet cells of 100 μm^2^ in five different sections were counted. The data are expressed as the means ± standard deviation (*n* = 10). *** *p* < 0.001 compared to control group. # *p* < 0.05 and ### *p* < 0.001 compared to the PM_2.5_-induced DED group.

To evaluate the population of goblet cells that secrete gel-forming mucins in the conjunctiva, we performed PAS staining. In control rats, a large number of violet PAS-positive goblet cells were observed in the conjunctival tissue (Figure 3A). However, topical exposure to PM_2.5_ greatly suppressed the frequency of PAS-stained goblet cells, but this was markedly enhanced following CFW 400 administration. Figure 3B shows the quantitative values of the conjunctival goblet cell population, and these are expressed as number per 100 μm^2^. The number of conjunctival goblet cells greatly reduced from 4.00 ± 0.82/100 μm^2^ to 0.26 ± 0.55/100 μm^2^ following exposure to PM_2.5_. Nevertheless, PM_2.5_-mediated conjunctival goblet cell loss was substantially recovered by oral administration of CFW in a dose-dependent manner. Furthermore, the density of conjunctival goblet cells in the CFW 400 group was higher than that in the CsA group. Meanwhile, oral administration of lutein did not improve conjunctival goblet cells loss. This result suggested that conjunctival goblet cell loss, a DED-mediated event, was markedly induced following exposure to PM_2.5_, and this alteration was significantly prevented by oral administration of CFW.

### 3.6. Effect of CFW on Inflammation of Lacrimal Gland in PM_2.5_-Induced DED Rats

**Figure 4:**
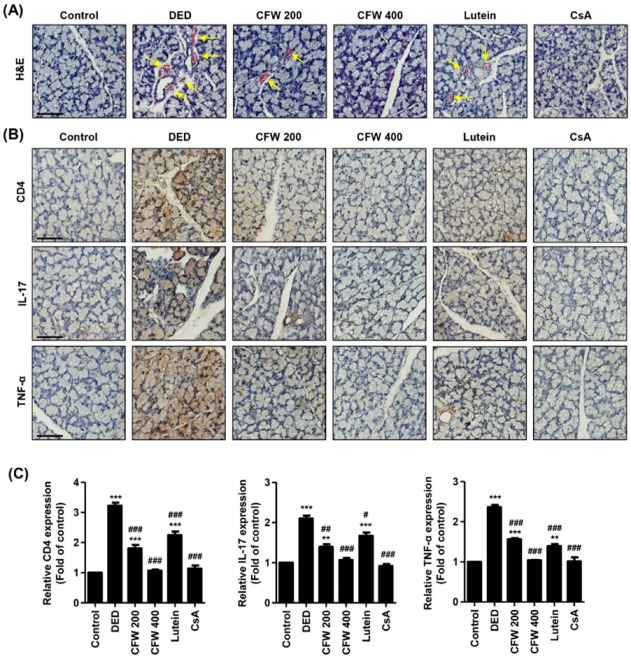
Effect of oral administration of CFW on inflammation of the lacrimal gland in PM_2.5_-induced DED rats. (**A**) Representative images of H&E staining in the lacrimal gland (*n* = 6). Yellow arrows indicate neo-vessels. Scale bar, 50 μm. (**B**) Representative images of immunohistochemical staining for CD4, IL-17, and TNF-α in lacrimal gland sections (*n* = 5). Scale bar, 50 μm. The brown-stained precipitates indicate the presence of the target antigen. (**C**) The stained area of the photograph was quantitative analyzed using ImageJ^®^ and calculated in terms of the fold of the control. ** *p* < 0.01 and *** *p* < 0.001 compared to the control group. The data are expressed as the means ± standard deviation (*n* = 3). # *p* < 0.05, ## *p* < 0.01 and ### *p* < 0.001 compared to the PM_2.5_-induced DED group.

We assessed the effect of CFW on the pathological changes in the lacrimal gland in rats with DED following exposure to PM_2.5_. Figure 4A shows normal secretory gland histology, including tight acini and ducts in control rats. However, exposure to PM_2.5_ led to inflammatory cell infiltration, sizable interstitial edema with abnormal acini, and the formation of neo-vessels around lobules. In contrast, oral administration of CFW prevented PM_2.5_-mediated histopathological changes in the lacrimal gland, in a dose-dependent manner. Meanwhile, lutein administration also inhibited infiltration of inflammatory cells and edema with abnormal acini, but neo-vessels around lobules were still slightly present. To further investigate whether CD4^+^ T cell immune responses are involved in PM_2.5_-mediated histopathological alteration of the lacrimal gland, we performed immunohistochemical staining for specific antibodies, such as CD4, IL-17, and TNF-α. The top panels of Figure 4B show that the predominant immune cell subset in the lacrimal glands of PM_2.5_-exposed rats consisted of CD4^+^ T cells. However, the overexpression of CD4^+^ T cells by PM_2.5_ was significantly down-regulated to control levels by oral administration of CFW 400 and cyclosporine (Figure 4B,C). In addition, the expression of IL-17 and TNF-α in the lesions of the lacrimal gland was significantly increased to 2.10- and 2.36-fold that of control by PM_2.5_ topical exposure. However, the upregulated expression was substantially attenuated following CFW 400 and cyclosporine treatment, and was similar to control levels following treatment. However, treatment was less effective on the expression of CD4, IL-17, and TNF-α in lutein group. These results suggest that PM_2.5_ exposure leads to pathological changes in the lacrimal gland, including inflammation, neovascularization, and abnormal acini, due to CD4^+^ T cell immune responses. Nevertheless, oral administration of CFW markedly prevented these alterations of the lacrimal gland by PM_2.5_, and the efficacy of spermidine was similar to that of cyclosporine treatment.

### 3.7. Effect of CFW on Histological Changes of the Retina after Topical Exposure to PM_2.5_ in SD Rats

**Figure 5:**
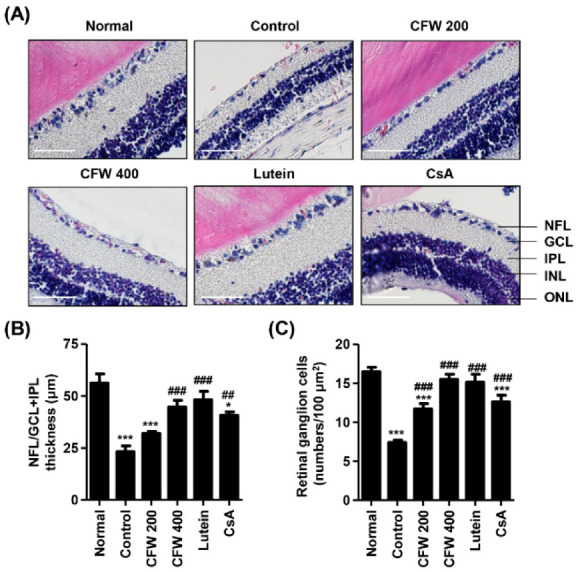
Effect of oral administration of CFW on retinal ganglion cell loss after topical exposure to PM_2.5_ in SD rats. (**A**) Representative images of cross-sectioned retina with H&E staining (*n* = 5). Nerve fiber layer (NFL), ganglion cell layer (GCL), inner plexiform layer (IPL), inner nuclear layer (INL), and outer nuclear layer (ONL) are indicated. Scale bar, 50 μm. (**B**) Thickness of NFL/GCL + IPL layers. (**C**) The numbers of cells in GCL of 100 μm^2^ in five different sections were counted. (**B**,**C**) The data are expressed as the means ± standard deviation (*n* = 10). * *p* < 0.05 and *** *p* < 0.001 compared to the normal group. ## *p* < 0.01 and ### *p* < 0.001 compared to the control group.

Next, we investigated the harmful effects on the retina following topical exposure to PM_2.5_, and the efficacy of CFW on PM_2.5_-mediated retinal alteration. As a result of H&E staining in the retinal section, the thickness of the nerve fiber layer (NFL), ganglion cell layer (GCL), and inner plexiform layer (IPL) markedly decreased by exposure to PM_2.5_, but this was markedly prevented by CFW 400, lutein, and CsA administration (Figure 5A,B). Meanwhile, the thickness of the inner nuclear layer (INL) and outer nuclear layer (ONL) did not differ among the groups. In addition, the population of ganglion cells in GCL was markedly decreased by exposure to topical PM_2.5_ (Figure 5A). However, the population of ganglion cells was greatly increased after CFW treatment. Figure 5C shows that the number of ganglion cells was significantly decreased in the retina of the DED group (7.42 ± 1.08/100 μm^2^) compared with that in the retina of the control group (16.50 ± 1.98/100 μm^2^). In contrast, oral administration of 200 mg/kg and 400 mg/kg CFW significantly enhanced the ganglion cell population to 11.71 ± 1.80/100 μm^2^ and 15.55 ± 1.93/100 μm^2^, respectively. The efficacy of CFW 400 on the improvement of ganglion cells was superior to that of cyclosporine, and similar to that of the lutein treatment.

## 4. Discussion

In this study, we investigated the effect of oral administration of CFW on the changes of the eyes, as well as on the hematology and biochemistry in a DED model induced by topical exposure to PM_2.5_. Interestingly, topical exposure to PM_2.5_ led to partial abnormality of the serum lipid profile, including TC and LDL-C. However, our findings showed that CFW contributes to the normalization of TC and LDL-C levels. Increasing epidemiological studies have recently shown that PM_2.5_ may have a hazardous influence on the metabolic system, including an increased risk of dyslipidemia [36,37]. One cohort study demonstrated that exposure to PM_2.5_ is associated with worsening LDL-C levels [36]. In this cohort study, researchers obtained atmospheric monitoring data for the daily mean concentration of hourly measured PM_2.5_ from the Ministry of Environment of Korea, and multiple regression analyses were conducted to assess the associations between exposure to PM_2.5_ and changes in lipid profiles at two-year intervals. Another cohort study investigated individual daily exposure to fine PM that was estimated by as spatiotemporal model, and analyzed the correlation between daily exposure levels of fine PM and blood lipid [37]. They suggested that high levels of fine PM exposure were closely correlated with increasing TC and LDL-C levels. Even though these few studies reported that exposure to ambient PM_2.5_ may have a negative effect on lipid profiles, such as TC and LDL-C, no studies have investigated the influence of topical exposure to PM_2.5_ on serum lipid profiles. In this context, our findings were very meaningful in that CFW treatment contributed to decrease serum TC and LDL-C levels that were markedly enhanced by topical exposure to PM_2.5._ Actually, several studies demonstrated that CF and its bioactive components improved dyslipidemia [38,39,40,41]. Park et al. [38] suggested that CF decreased serum TC levels with a suppress in esterified cholesterol in hypercholesterolemic rat. In addition, Zhang et al. [39] demonstrated that polysaccharide isolated from CF attenuated the cholesterol accumulation in oxidized LDL-stimulated macrophages and suppressed LDL-C, TC, and TG levels in high-fat-diet-fed mice. Furthermore, it has been reported that 7-O-galloyl-D-sedoheptulose, isolated from CF, improved the levels of lipid profile in type 2 diabetic mice [40]. Another study demonstrated that oral administration of iridoid glycoside from CF regulated lipid metabolism in db/db mice [41].

The ocular surface is composed of the corneal and conjunctival epithelium, corneoscleral limbus, nerves, and tear film [42]. The ocular surface serves as a barrier to chemicals, microbes, and other airborne matter and provides anatomic, physiologic, and immunologic protective functions [42,43]. Accumulated epidemiological and biochemical studies demonstrate that exposure to PM_2.5_ may contribute to DED, which is a multifactorial ocular surface disease that involves tear film instability, hyperosmolarity, inflammation, and damage to the ocular surface [6,8,9,11,12]. The tear film is composed of three distinct layers, a mucin layer, an aqueous layer, and a lipid layer, and performs many physiological functions [44]. Compromise of the tear film triggers tear film destabilization, promotes exposure of the corneal epithelium to air, and potentially contributes to dry eye symptoms [45]. A mucin layer composed of mucins produced by conjunctival epithelial cells provides an easily wettable ocular surface and assists in tear re-spreading after blinking [46,47]. The conjunctival epithelium houses mucin-producing goblet cells [48]. Recently, several animal studies have demonstrated that topical administration of PM_2.5_ presented dry eye phenotypes, accompanied by a decreased tear production, damaged corneal epithelium, reduced conjunctival goblet cells, and an abnormal corneal structure [5,12,13,35]. In this context, the clinical characteristic of DED can be diagnosed the tear break up time (TBUT), the tear secretion test and goblet cell counting [5]. In this study, we also reproduced a DED murine model induced by topical instillation of PM_2.5_, which was accompanied by decreased tear secretion, induced detachment of the corneal epithelium, and loss of conjunctival goblet cells. However, oral administration of CFW significantly protected tear reduction, corneal epithelial detachment, and conjunctival goblet cell loss following PM_2.5_ exposure. Recently, a newly discovered DED subtype is the short TBUT that is related with ocular neuropathic pain and eye strain [49], The TBUT analysis was developed to judge the abnormalities of the tear film [50]. Actually, several previous studies reported that PM_2.5_ markedly decreased TBUT in DED murine models [5,12,13,35]. Although our current result suggested that CFW treatment involved in increment of tear production, the further studies are need to verify the efficacy of CFW on TBUT in PM_2.5_-exposed DED model. Nevertheless, our present finding showed that recovery of conjunctival goblet cells by oral administration of CFW may contribute to the stabilization of the mucin layer and lead to tear film stability. Therefore, this finding supported that oral application of CFW increased tear production and induced tear film stability through reinforcement of the mucin layer and this was ultimately caused by ocular surface stability.

The major pathogenesis of DED is an inflammation, including infiltrated immune cells in the conjunctiva and lacrimal glands, increased density of dendritic cells in the cornea, and increased secretion levels of tear cytokines [51,52]. Inflammation of the ocular surface in DED is sustained by the ongoing activation and infiltration of pathogenic immune cells, primarily of CD4^+^ T cells [53]. The main proliferating subset of dry eye T cell effectors in the presence of T regulatory cells is IL-17-secreting CD4^+^ T cells [54]. IL-17 induces the secretion of pro-inflammatory cytokines such as IL-1, IL-6, and TNF-α, and these cytokines are upregulated in DED [55]. In this regard, one study reported that the topical application of PM_2.5_ elevated dendritic cell maturation and the expression of TNF-α, IL-1β, and IL-6 in a murine model [56]. Another study also reported that the expression of TNF-α and IL-6 was increased in the PM_2.5_-induced DED rat [57]. Although the role of CD4^+^ T cell-mediated immune responses in DED is well-established, there are no studies on the relationship between CD4^+^ T cell-mediated immune responses in PM_2.5_-induced DED. Just one study verified that PM_2.5_-stimulated CD4^+^ T cells potently increased the mRNA and protein levels of pro-inflammatory cytokines and induced the death of human bronchial epithelial cells [58]. Herein, we found that topical exposure to PM_2.5_ was caused by the pathological changes in the lacrimal gland, including inflammation, neovascularization, and abnormal acini. Additionally, PM_2.5_ increased the expression of CD4^+^ T cells, IL-17, and TNF-α in the lacrimal gland. However, oral administration of 400 mg/kg CFW markedly protected these PM_2.5_-mediated inflammatory responses of the lacrimal gland, and the efficacy of CFW was similar to that of cyclosporine treatment and superior to that of lutein. These results indicate that oral supplements of CFW may contribute to the suppression of CD4^+^ T cell immune responses induced by PM_2.5_. Furthermore, we considered that the protective effect of CFW on lacrimal gland inflammation in PM_2.5_-exposed rats was due to its anti-inflammatory potential [22,27].

The blood–retina barrier (BRB) is available for diffusion and permeabilization of PM_2.5_, and its function in BRB could potentially play a role in PM_2.5_-mediated retinal pathogenesis [59,60]. Several epidemiological studies have suggested that exposure to PM_2.5_ correlates with retinal damage, including retinal atherosclerosis, retinal edema, and retinal vessel narrowing [59,60,61]. One study demonstrated that topical exposure to PM_2.5_ markedly decreased the NFL/GCL and increased the expression of glial fibrillary acidic protein [4]. Furthermore, a correlation between PM_2.5_ and changes in retinal structure features in subjects exposed to higher levels of PM_2.5_ was reported [59]. Previously, we suggested that PM_2.5_ mediates retinal dysfunction through ROS-mediated epithelial–mesenchymal transition and necrotic and autophagic cell death in retinal pigment epithelial cells [62,63]. Our present findings showed that topical exposure to PM_2.5_ led to the loss of retinal ganglion cells and the decrease of the NFL/GCL + IPL thickness; however, oral administration of CFW contributed to the normalization of retinal ganglion cell density and retinal constitution.

In the present study, we used two positive controls: CsA and lutein. CsA is a T cell immunomodulatory agent that is used to suppress ocular surface inflammation and improve tear film dynamics [32,33]. To date, the effect of CsA on PM_2.5_-exposed eyes is yet to be reported. Our results showed that CsA attenuated the DED symptoms, including decreasing tear secretion, epithelium detachment, loss of conjunctival goblet cells, and inflammation of lacrimal gland following topical exposure to PM_2.5_. In addition, CsA improved serum TC levels and restored retinal ganglion cell population and NFL/GCL + IPL thickness, which is the first finding for the effect of CsA on the posterior segment of the eye following exposure to PM_2.5_. However, this efficacy of CsA is less or similar than that of CFW. It is also noteworthy that CsA is an immunosuppressant drug widely used oral medications in organ transplant recipients and patients with autoimmune disorders. Some studies reported that long-term treatment with oral administration of CsA was associated with hyperlipidemia and an increased risk of atherosclerosis, but the mechanisms by which cyclosporin A causes hyperlipidemia are unclear [64,65]. However, these previous reports about the side effects of oral CsA administration are contrary with our present finding. The differences between previous study and the present study are the administered period and route. Therefore, we consider that further studies are needed to verify whether improvement of TC levels by CsA eye drops is a temporary situation, and to evaluate the effect of long-term topical application of CsA.

Meanwhile, lutein is one of the xanthophyll carotenoids and has posterior eye-protective properties through anti-oxidative and anti-inflammatory effects [31]. Numerous clinical studies suggested that lutein supplements reduce neovascular age-related macular degeneration (AMD) risk, improve visual function, and increase macular pigment optical density [66,67,68]. However, the effect of lutein on PM_2.5_-exposed eyes, especially ocular surface, is yet to be reported. In the present study, we found that lutein had slightly improved efficacy or no efficacy on the anterior segment of eye in PM_2.5_-exposed rat, but very superior efficacy on the posterior segment of eye. Oral administration of lutein caused a minor increase in tear secretion and corneal epithelium stabilization, and inhibited inflammation of the lacrimal gland, while it had no effect on the conjunctival goblet cell loss. On the other hand, the lutein supplement markedly increased the retinal ganglion cell population and NFL/GCL + IPL thickness following exposure to PM_2.5_. Interestingly, the efficacy of CFW on the retina was similar to that of lutein. Overall, in the present study, we found that oral administration of CFW protected against PM_2.5_-induced DED symptoms, retinal disorder, and dyslipidemia. More importantly, the efficacy of CFW was superior and/or similar to that of cyclosporine A, an anti-inflammatory agent, and lutein, the posterior eye-protective agent.

Numerous reports demonstrated that women are disproportionately affected by DED, are diagnosed at a younger age, and experience more severe symptoms compared with men [69,70]. Similar to the human studies, several animal studies suggested female sex is a risk factor for DED [5,71,72]. McClellan et al. [71] reported that female C57BL/6 mice developed greater corneal barrier disruption than age-matched male mice did, although other features of DED such as low goblet cell density and LG infiltration were similar in both sexes. Recently, Song et al. [72] used six-week-old female SD rats to assess the effect of natural products on a PM_2.5_-induced DED model. Yang et al. [5] also used 6–8-week-old female C57BL/6 mice to evaluated the harmful effect of PM_2.5_ on the eye. Based on this knowledge, we demonstrated that CFW prevented PM_2.5_-indued DED in female SD rats. Although we assessed the effect of CFW on PM_2.5_-exposed female SD rats, we consider that further studies are needed to evaluate the influence of topical exposure to PM_2.5_, and to confirm the efficacy of CFW in males.

## 5. Conclusions

Taken together, our present findings indicated that oral administration of CFW contribute to normalize the levels of serum TC and LDL-C in the topical PM_2.5_-exposed rat. Furthermore, oral administration of CFW protected tear film destabilization, inflammation of the lacrimal gland, and histological changes in the retinal NFL/GCL and IPL in the PM_2.5_-induced DED. Therefore, the present findings may provide an experimental basis for the potential application of CFW in preventing air pollution-related dry eye symptoms, retinal disorders, and lipid metabolism disorder.

## Figures and Tables

**Table 1:** Effect of oral administration of CFW on the changes of the body weight and organ weight in PM_2.5_-exposed SD rats.

Organ Weight (g)	Group					
	Control	DED	CFW 200	CFW 400	Lutein	CsA
BW gain	31.92 ± 9.35	28.11 ± 9.03	31.13 ± 6.94	30.05 ± 8.07	30.48 ± 6.77	34.03 ± 10.04
Thymus	0.40 ± 0.08	0.39 ± 0.07	0.41 ± 0.06	0.42 ± 0.10	0.36 ± 0.04	0.38 ± 0.09
Heart	0.69 ± 0.04	0.68 ± 0.03	0.67 ± 0.04	0.65 ± 0.06	0.66 ± 0.02	0.67 ± 0.06
Lung	1.08 ± 0.09	1.02 ± 0.07	1.01 ±0.06	0.98 ±0.08	0.99 ± 0.06	1.01 ± 0.07
Liver	6.43 ± 0.47	6.47 ± 0.59	6.33 ± 0.68	6.28 ± 0.59	6.42 ± 0.88	6.44 ± 0.67
Kidney	1.52 ± 0.18	1.47± 0.11	1.49 ± 0.09	1.51 ± 0.14	1.45 ± 0.06	1.49 ± 0.12
Spleen	0.53 ± 0.04	0.52 ± 0.07	0.52 ± 0.07	0.53 ± 0.05	0.51 ± 0.08	0.54 ± 0.04
Uterus and Ovary	0.51 ± 0.10	0.48 ± 0.08	0.49 ± 0.11	0.52 ± 0.10	0.59 ± 0.18	0.58 ± 0.14

The data are expressed as mean ± standard deviation (*n* = 5). Control, untreated group; DED, PM_2.5_ with normal saline-treated group; CFW 200, PM_2.5_ with 200 mg/kg of water extracts of Corni Fructus-treated group; CFW 400, PM_2.5_ with 400 mg/kg of water extracts of Corni Fructus-treated group; Lutein, PM_2.5_ with lutein-treated group; and CsA, PM_2.5_ with cyclosporine A-treated group. CsA, cyclosporin A.

**Table 2:** Effect of oral administration of CFW on the hematological, biochemical, and lipid profiles in SD rats after topical exposure to PM_2.5_.

	Group					
	Control	DED	CFW 200	CFW 400	Lutein	CsA
RBC (10^6^/μL)	8.15 ± 0.16	8.20 ± 0.12	8.11 ± 0.24	8.03± 0.35	8.06 ± 0.15	7.91 ± 0.30
WBC (10^3^/μL)	5.10 ± 0.73	4.55 ± 1.23	4.82 ± 0.86	4.32 ± 0.98	4.41 ± 1.11	4.09 ± 1.37
Hematocrit (%)	50.04 ± 1.65	49.58 ± 1.25	49.96 ± 2.08	49.42 ± 2.65	50.56 ± 1.24	49.62 ± 2.21
Hemoglobin(g/dL)	15.46 ± 0.21	15.32 ± 0.17	15.25 ± 0.82	15.08 ± 0.95	15.06 ± 0.69	15.04 ± 0.51
MCV (fL)	61.42 ± 1.71	60.32 ± 0.97	60.87 ± 1.11	61.52 ± 1.40	61.20 ± 0.82	61.20 ± 2.24
MCH (pg)	19.00 ± 0.32	18.72 ± 0.34	18.89 ± 0.63	18.76 ± 0.62	19.04 ± 0.45	19.20 ± 0.71
MCHC (g/dL)	30.98 ± 0.57	31.02 ± 0.27	30.44 ± 0.61	30.50 ± 0.44	30.38 ± 0.73	30.96 ± 0.47
Platelet (10^3^/μL)	839.60 ± 81.15	912.67 ± 63.07	885.71 ± 54.83	873.40 ± 62.96	870.80 ± 45.92	898.00 ± 48.46
AST (U/L)	141.26 ± 24.35	150.88 ± 20.75	142.18 ± 19.48	138.04 ± 23.77	143.28 ± 15.23	139.30 ± 21.43
ALT (U/L)	20.94 ± 2.49	22.93 ± 3.68	21.53 ± 3.05	22.48 ± 4.14	21.42 ± 1.57	20.54 ± 2.55
ALP (U/L)	428.24 ± 41.58	428.87 ± 63.12	431.17 ± 48.49	421.12 ± 62.66	439.28 ± 38.65	428.76 ± 79.26
BUN (mg/dL)	14.19 ± 0.94	14.40 ± 1.15	14.38 ± 1.02	14.89 ± 0.75	14.22 ± 1.66	14.43 ± 1.30
Creatinine (mg/dL)	0.48 ± 0.03	0.48 ± 0.04	0.48 ± 0.02	0.47 ± 0.03	0.48 ± 0.02	0.48 ± 0.03
TC (mg/dL)	57.98 ± 3.37	74.47 ± 5.95 **	66.18 ± 3.35	62.20 ± 3.77 ^#^	65.38 ± 8.59	60.65 ± 4.17 ^#^
TG (mg/dL)	48.44 ± 5.13	48.00 ± 7.94	45.77 ± 5.85	43.64 ± 6.62	45.26 ± 8.72	46.58 ± 8.63
HDL-C (mg/dL)	29.50 ± 3.26	28.35 ± 3.97	28.97 ± 3.49	29.18 ± 3.32	28.56 ± 3.33	29.32 ± 4.25
LDL-C (mg/dL)	6.08 ± 0.39	9.68 ± 1.28 ***	7.78 ± 0.65 ^#^	7.18 ± 0.91 ^##^	8.45 ± 0.21 **	8.47 ± 0.90 **
FFA (uEq/L)	665.00 ± 27.81	720.17 ± 82.88	688.61 ± 49.04	683.60 ± 43.76	676.20 ± 102.10	695.00 ± 56.31

The data are expressed as mean ± standard deviation (*n* = 5). ** *p* < 0.01 and *** *p* < 0.001 compared to the control group. ^#^ *p* < 0.05 and ^##^ *p* < 0.01 compared to PM_2.5_-induced DED group. RBC, red blood cell; WBC, white blood cell; MCV, mean corpuscular volume; MCH, mean corpuscular hemoglobin; MCHC, MCH concentration; AST, aspartate aminotransferase; ALT, alanine aminotransferase; ALP, alkaline phosphatase; BUN, blood urea nitrogen; TC, total cholesterol; TG, triglyceride; HDL-C, high-density lipoprotein cholesterol; LDL, low-density lipoprotein cholesterol; FFA, free fatty acid.

## Data Availability

The datasets during and/or analyzed during the current study are available from the corresponding author on reasonable request.
